# Alcohol-mediated susceptibility to lung fibrosis is associated with group 2 innate lymphoid cells in mice

**DOI:** 10.3389/fimmu.2023.1178498

**Published:** 2023-06-29

**Authors:** Liang Chen, Rui Sun, Chao Lei, Zhishan Xu, Yong Song, Zhongbin Deng

**Affiliations:** ^1^ Department of Surgery, Division of Immunotherapy, University of Louisville, Louisville, KY, United States; ^2^ Department of Respiratory and Critical Care Medicine, The Affiliated Huaian No. 1 People’s Hospital, Nanjing Medical University, Huai’an, Jiangsu, China; ^3^ Brown Cancer Center, University of Louisville, Louisville, KY, United States; ^4^ Department of Respiratory Medicine, Jinling Hospital, Nanjing University School of Medicine, Nanjing, Jiangsu, China

**Keywords:** alcohol, lung fibrosis, neutrophils, ILC2, neuroimmune, neuropeptide, CGRP

## Abstract

Chronic alcohol ingestion promotes acute lung injury and impairs immune function. However, the mechanisms involved are incompletely understood. Here, we show that alcohol feeding enhances bleomycin-induced lung fibrosis and inflammation via the regulation of type 2 innate immune responses, especially by group 2 innate lymphoid cells (ILC2s). Neuroimmune interactions have emerged as critical modulators of lung inflammation. We found alcohol consumption induced the accumulation of ILC2 and reduced the production of the neuropeptide calcitonin gene-related peptide (CGRP), primarily released from sensory nerves and pulmonary neuroendocrine cells (PNECs). CGRP potently suppressed alcohol-driven type 2 cytokine signals *in vivo*. Vagal ganglia TRPV1^+^ afferents mediated immunosuppression occurs through the release of CGRP. Inactivation of the TRPV1 receptor enhanced bleomycin-induced fibrosis. In addition, mice lacking the CGRP receptor had the increased lung inflammation and fibrosis and type 2 cytokine production as well as exaggerated responses to alcohol feeding. Together, these data indicate that alcohol consumption regulates the interaction of CGRP and ILC2, which is a critical contributor of lung inflammation and fibrosis.

## Introduction

The most common health problems associated with alcohol use disorder (AUD) include liver steatosis and cirrhosis (1, 2), cardiomyopathies (3) and nerve damage (i.e., neuropathies) (4). Recent evidence indicates that the lung is also adversely affected by alcohol abuse. Chronic alcohol ingestion may render the lung susceptible to fibrosis in the mouse model of bleomycin-induced acute lung injury (ALI) (5), pulmonary infections (6), and acute respiratory distress syndrome (ARDS) (7). Lung inflammatory cytology is thought to contribute to fibrogenesis, including neutrophils, lymphocytes, and macrophages (8, 9). Alcohol consumption can induce dysregulation of neutrophil recruitment and change the functioning of T helper 17 (Th17) cells in alcohol-related liver disease (ALD) (10). Although chronic alcohol consumption is clearly linked to an increased risk of ALI, the role that alcohol plays in the pathogenesis of pulmonary fibrosis is not fully understood.

Type 2 cytokines, such as TGF-β1, interleukin-13 (IL-13) and IL-5, are essential for the development of lung fibrosis by promoting production of extracellular matrix proteins and inducing myofibroblast differentiation (9). These type 2 cytokines rely on tightly coordinated and regulated immune responses across different cell types, such as T helper 2 (Th2) cells, mast cells, and ILC2s. ILC2s are primarily found at mucosal surfaces and have emerged as key regulators of type 2 inflammation (11, 12). Recent studies have highlighted the role of ILC2s in the development of allergic diseases, e.g. airway hyperreactivity (13), and liver (14) and pulmonary fibrosis (9). ILC2s respond to alarmins signals, from the tissue microenvironment, i.e., IL-25, or IL-33, which are released by epithelial cells upon stress or damage (15, 16). Many factors have been shown to either negatively or positively regulate ILC2 function, including cytokines (17, 18), cell surface receptors (19), and lipid mediators (20, 21). However, whether and how alcohol ingestion induces quantitative and/or functional changes to lung ILC2 is not well examined. We hypothesized that increased susceptibility to bleomycin-induced ALI could be caused by alcohol-induced stress and dysregulation of ILC2 immune responses.

Chronic alcohol administration can cause the neurodegeneration in the central nervous system (CNS) (22). Our recent study revealed that ethanol exposure reduces the numbers of enteric vasoactive intestinal polypeptide (Vip)-producing neurons and inhibits the neuropeptide Vip secretion from the enteric neuron *in vivo* and *in vitro* (23). Neuron-immune interactions are critical for regulating ILC2s function in mucosal tissues. For example, in the mouse gastrointestinal tract, ILC2s co-localize with cholinergic neurons that express the neuropeptide neuromedin U (NMU) and selectively express the NMU receptor 1 (NMUR1) (24, 25). Acting with the IL-25 and IL-33, NMU induces ILC2 proliferation, increases secretion of the type 2 cytokines and lung inflammation, and promotes the expulsion of the gastrointestinal nematode *Nippostrongylus brasiliensis* (26). α-CGRP (α-calcitonin gene-related peptide) is a 37-amino-acid neuropeptide produced as an alternatively spliced product of the calcitonin (*Calca*) gene (27) and secreted by pulmonary neuroendocrine cells (PNECs) in the lung (28). CGRP was reported to regulate the ILC2 response in the lung (29) and intestine (30). CGRP-producing transient receptor potential vanilloid (TRPV)1^+^ neurons attenuates severity of *Staphylococcus aureus*-induced pneumonia by limiting γδ T cells and neutrophils in the lung (31). Nevertheless, it is unclear whether alcohol consumption contributes to the production of neuropeptide CGRP and the regulation of lung ILC2 in ALI.

Here, we used a well-characterized mouse model of bleomycin-induced ALI to evaluate the effects of chronic alcohol ingestion on pulmonary fibrosis. To uncover key responding cellular components, we profiled immune cells from lung in the chronic alcohol consumption and/or its related ALI model. Our data showed that chronic alcohol ingestion regulates lung KLRG1^+^ ILC2-related type 2 immunity. We identified the neuroimmune pathway that modulates ILC2s responses by analyzing expression of both neuropeptides and their receptors and demonstrated that CGRP-Ramp1 signaling contributes to the lung fibrosis via ILC2s in the model of alcohol primed bleomycin-induced lung injury.

## Results

### Alcohol enhances bleomycin‐induced lung inflammation and fibrosis

**Figure 1:**
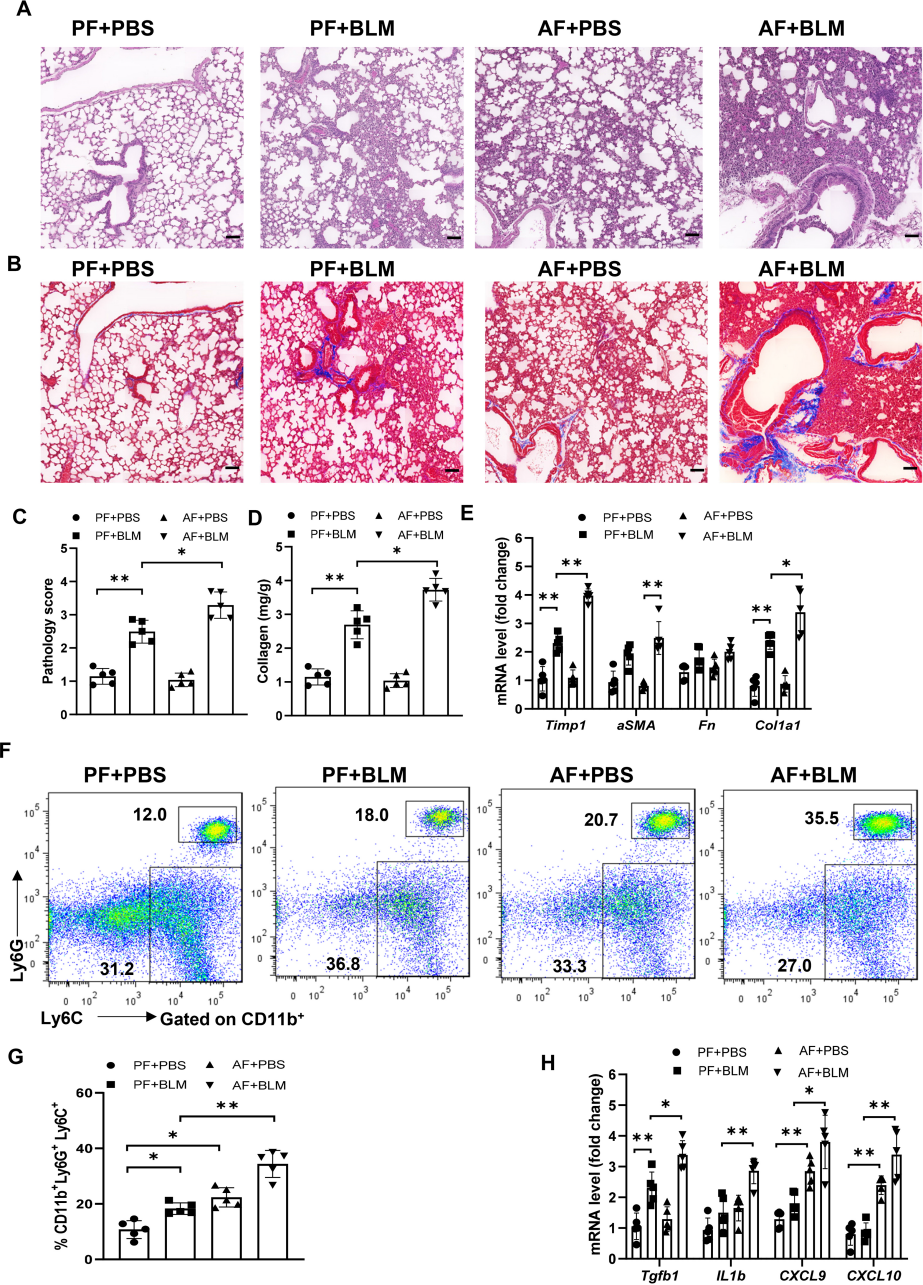
Chronic alcohol ingestion promotes bleomycin-induced lung injury. 8-week-old mice were fed control diet (PF) or alcohol diet (AF) for 14 days and then treated with bleomycin. Mice were continually on control diet or alcohol diet. Lungs were harvested and analyzed at 14 days after bleomycin or PBS challenge. **(A, B)** Hematoxylin and eosin **(A)** and Masson’s trichrome staining **(B)**. Scale bar: 50µM. **(C)** Lung pathology score. **(D)** Lung tissue collagen content. **(E)** mRNA levels of the indicated genes related to fibrosis in lung. Fn: Fibronectin. **(F, G)** Frequency of lung neutrophils (CD11b^+^Ly6G^+^Ly6C^+^) and monocytes (CD11b^+^Ly6G^-^Ly6C^+^), determined by flow cytometry. **(H)** mRNA levels of the indicated genes in lung. Representative of images from independent experiments (**A** and **B**, n=5); Error bars Mean ± SEM; n =5 **(C-H)**, *p < 0.05, **p < 0.01.

To examine if the alcohol ingestion affects lung immune function in ALI, we fed an alcohol (alcohol feeding [AF]) or an isocaloric control (pair feeding [PF]) diet to male and female C57BL/6 mice (10). Mice were administrated with bleomycin to induce ALI at day 14 after alcohol feeding and continued on the control or alcohol diet for 14 days. The lungs from alcohol-fed mice with bleomycin-treatment showed evidence of increased fibrosis as reflected by Masson’s trichrome staining and collagen deposition when compared to those from either control diet-fed mice treated with bleomycin or to control diet-fed or alcohol diet-fed mice (**Figures 1A–D**). The levels of lung fibrosis markers, including Timp1, collagen type I alpha 1 chain (Col1a1), and alpha-smooth muscle actin (α-SMA) mRNAs, were highest in bleomycin-treated alcohol-fed mice (**Figure 1E**). Flow cytometry and immunohistochemistry analysis revealed that alcohol feeding induced the massive infiltration of CD11b^+^Ly6G^+^Ly6C^+^ neutrophils into the lung (**Figures 1F, G**, **Figure S1A**). Interestingly, alcohol consumption reduced the levels of CD11b^intermediate^ F4/80^-^ myeloid cells (**Figure S1B**). Real-time PCR results revealed that amounts of TGFβ, chemokine (C-X-C motif) ligand 9 and 10 (CXCL9 and CXCL10), and IL-1β mRNA in the lung were increased in bleomycin-treated alcohol-fed mice (**Figure 1H**). These combined data indicate that alcohol consumption in the acute bleomycin injury model promotes pulmonary immune cell infiltration and collagen accumulation and increases histopathological changes in the lungs. Thus, chronic alcohol ingestion regulates key lung parameters related to inflammation and fibrosis.

### Chronic alcohol ingestion induces lung ILC2s-mediated type 2 immunity

**Figure 2:**
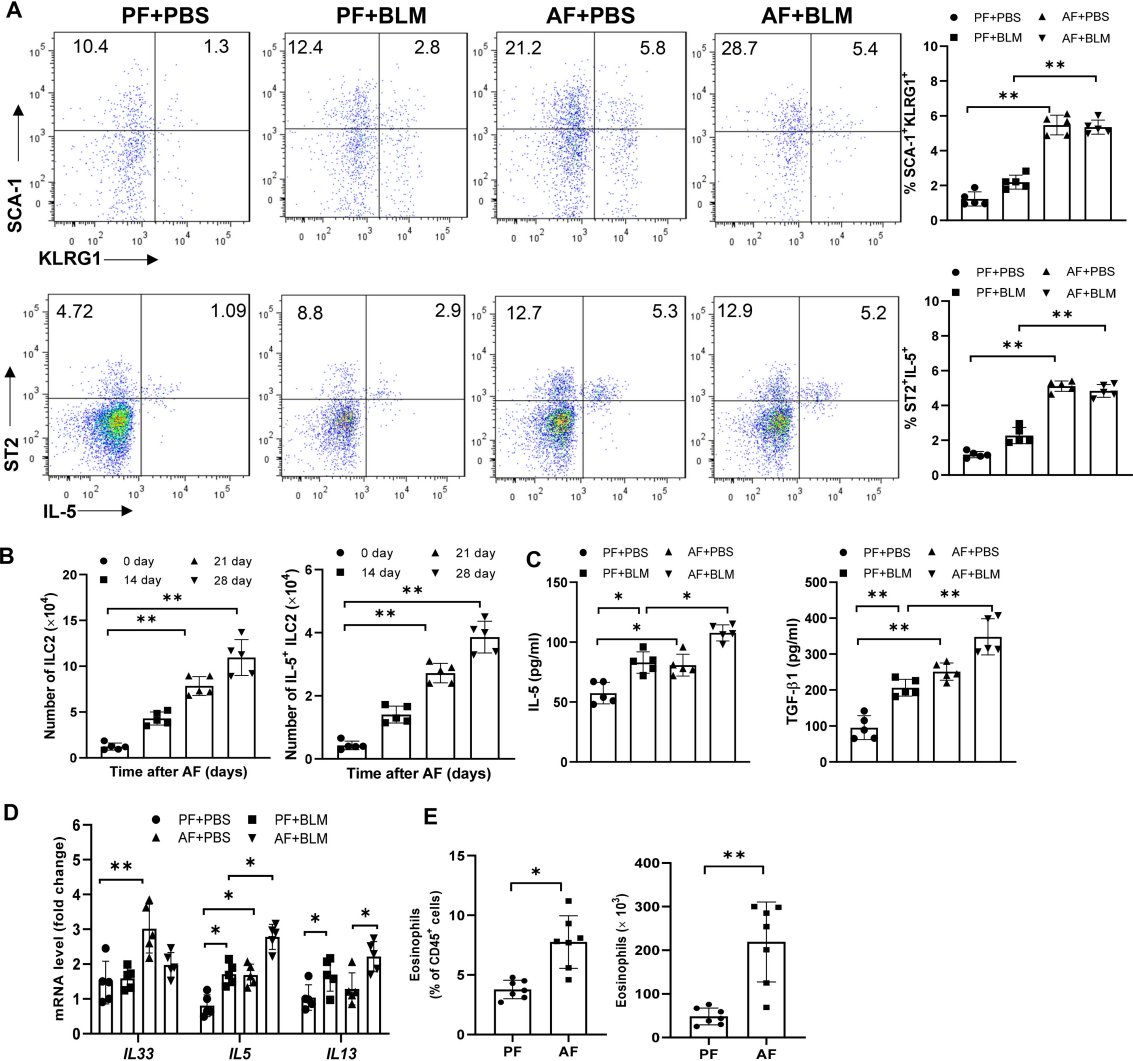
Alcohol feeding induces an ILC2s-related type-2 immune response in the lung. 8-week-old mice were fed control diet (PF) or alcohol diet (AF) for 14 days and then treated with bleomycin. Mice were continually on control diet or alcohol diet. Lungs were harvested and analyzed at 14 days after bleomycin or PBS challenge. **(A, B)** The frequency **(A)** and number **(B)** of KLRG1^+^ ILC2s and IL-5^+^ ILC2s in whole lung tissues, determined by flow cytometry. **(C)** IL-5 and TGF-β concentration in lung tissue determined by Multiplex and ELISA, respectively. **(D)** IL-33, IL-5 and IL-13 mRNA expression in lung leukocytes, determined by qRT-PCR. **(E)** The frequency (left) and number (right) of eosinophils (CD11c^−^ CD11b^+^ Siglec-F^+^) in whole lung tissues, determined by flow cytometry. n=5, *p < 0.05, **p < 0.01.

We next determined the kinetics of ILC2s levels in the lungs of alcohol-fed mice. We defined ILC2s as hematopoietic lineage marker (CD3, CD5, B220, CD11C, CD11b, Gr-1, and Ter-119)-negative, and positive for CD45, SCA1, ST2, KLRG1 and CD127 using flow cytometry. Eight-week-old WT mice were fed the alcohol diet and their lungs were harvested on days 0, 14, 21, and 28 for detection of ILC2s. At day 14 after alcohol ingestion, we noted a significant increase in the percentage of lung cells that were KLRG1^+^ and IL-5^+^ ILC2s, and the total number of KLRG1^+^ and IL-5^+^ ILC2s in alcohol-fed mice relative to control diet-fed mice (**Figures 2A, B**). However, lung ILC2s were not further increased in bleomycin-treated alcohol-fed mice (**Figure 2A**). Furthermore, we examined the changes of Th2 cells by analyzing the expression of GATA3, IL-5, IL-10 in CD3^+^ TCRβ^+^ CD4^+^ T cells and found Th2 cells were also increased by alcohol feeding, but not by bleomycin treatment (**Figures S2A, B**). In addition, we detected a slight increase of CD4^+^IL-17A^+^ Th17 cells and a similar proportion of CD4^+^Foxp3^+^ T regulatory cells (Tregs) and CD4^+^IFN-γ^+^ Th1 cells in AF mice (**Figure S2C**). Thus, administration of alcohol initially stimulated ILC2s, resulting in type 2 cytokine production in the lung. Compared with control diet, feeding with alcohol diet significantly increased IL-5 and TGF-β, but not IL-33, IL-13 and soluble ST2 protein concentration in lung homogenates (**Figure 2C**, **Figure S2D**). However, we found alcohol-fed mice have higher levels of IL*-*5, IL-13 and IL-33 transcripts in lung leukocytes (**Figure 2D**), and increased eosinophil numbers in the lung (**Figure 2E**).

### ILC2s are critical for chronic alcohol ingestion enhanced pulmonary fibrosis

**Figure 3:**
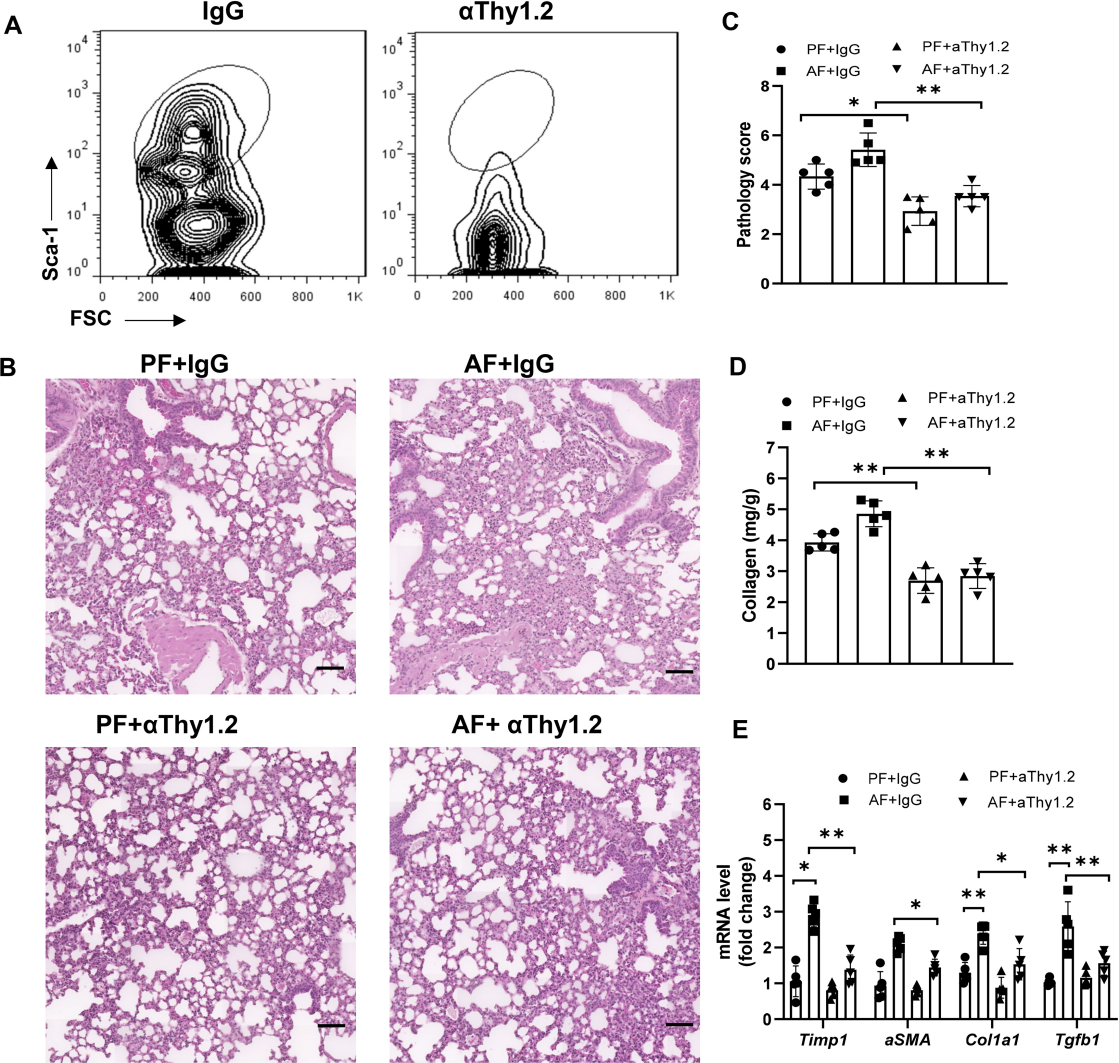
Alcohol-induced ILC2 promotes bleomycin-induced lung injury. Rag1^-/-^ mice were fed the control diet or alcohol diet and then treated with bleomycin for 14 days. Each group of mice were also received 200 μg of anti-Thy1.2 antibody or isotype control antibody every 3 days after bleomycin treatment. **(A)** The depletion efficiency of ILC2s (CD45^+^lin^−^CD127^+^CD90^+^ST2^+^CD25^+^) was determined by flow cytometry. The row was gated on CD45^+^CD127^+^lin^−^CD90^+^ live cells. **(B)** Hematoxylin and eosin staining. Scale bar: 50µM. **(C)** Lung pathology score. **(D)** Lung tissue collagen content. **(E)** mRNA levels of the fibrotic genes in lung, measured by qRT-PCR. Error bars Mean ± SEM; n=5, *p < 0.05, **p < 0.01.

To further determine whether the effect of alcohol ingestion on lung injury was dependent on ILC2s, total ILCs were depleted with anti-Thy1.2 antibody in bleomycin-induced Rag1^-/-^ mice. As expected, lung ILCs were effectively depleted (**Figure 3A**). Mice treated with anti-Thy1.2 antibody, particularly under an alcohol-fed condition, developed less inflammation and fibrosis than did the isotype-treated mice, as evidenced by the H&E staining of lung tissues (**Figures 3B, C**), collagen deposition (**Figure 3D**) and the decreased expression of fibrotic genes (**Figure 3E**). Taken together, these data suggest that alcohol mediated activation of ILC2s may mediate the development of lung fibrosis.

### Chronic alcohol ingestion suppresses the release of neuropeptide CGRP

**Figure 4:**
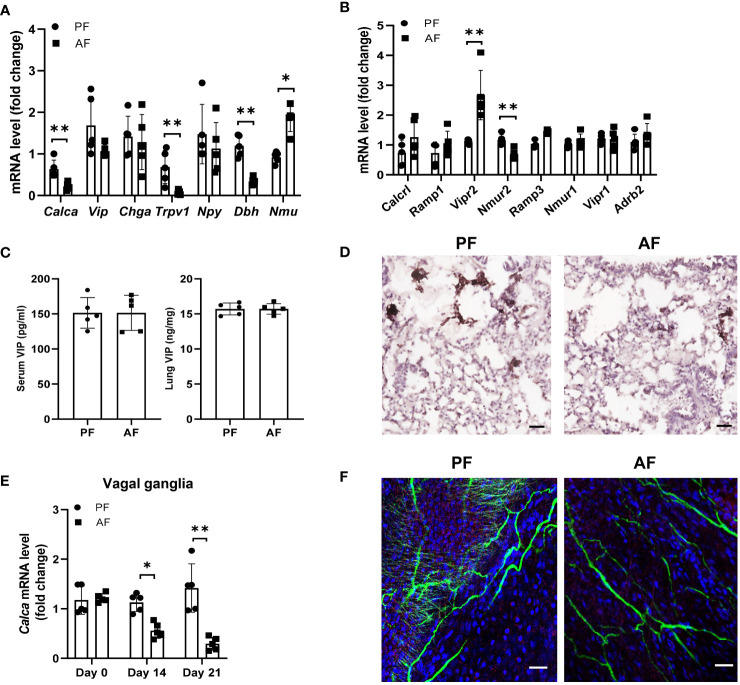
Chronic alcohol consumption suppresses the release of neuropeptide CGRP. Mice were fed the paired diet (PF) or alcohol diet (AF) for 21 days. **(A, B)** mRNA levels of genes encoding neuropeptides in lung **(A)** and the receptors for neuropeptides in lung ILC **(B)**, measured by qRT–PCR. **(C)** Enzyme immunoassay (EIA) measurement of concentration of VIP in the lung and serum of mice after alcohol feeding. **(D)** Immunohistochemistry analysis of CGRP^+^ PNECs. Scale bar: 50µM. **(E)** qRT-PCR of vagal ganglia Calca expression in mice at day 0, 14 and 21 after the start of alcohol challenge. **(F)** Representative whole mount images and image quantification of CGRP^+^ neurons by markers staining with βIII-Tub (green), CGRP (red), and DAPI (blue) in the trachea. Scale bar: 50µM. Error bars Mean ± SEM; n =5-7, **p < 0.01, representative of 3 independent experiments (**D**, **F**).

Alcoholic neuropathy is associated with altered neuronal activity and nerve damage (32, 33). Neuron-immune interactions are critical for regulating ILC2 function in mucosal tissues. To test the hypothesis that alcohol ingestion affects type 2 responses through neuroimmune interactions, we examined the expression of genes encoding several neuropeptides including *Calca* (CGRP), *Chga* (chromogranin A), *Npy* (neuropeptide Y), *Vip*, and *Nmu* (Neuromedin U) (34, 35). *Calca* mRNA levels, but not *Vip*, *Chga* and *Npy*, was greatly reduced after administration of alcohol (**Figure 4A**). However, NMU expression, which promotes ILC2 activation and amplifies allergic inflammation (25), was upregulated upon alcohol feeding. Activation of the nerve fibers followed by CGRP release is mediated by direct activation of the ion channel TRPV1 (36, 37). In alcohol-stimulated lungs, TRPV1 expression was significantly lower than in the lungs of control mice (**Figure 4A**). We also examined the expression of Dbh (Dopamine beta-hydroxylase), which catalyzes the conversion of dopamine to norepinephrine, and found alcohol feeding reduced the Dbh expression in lung tissues. We further tested the effect of alcohol consumption on neuropeptide receptors on ILC2s including CGRP co-receptor genes Calcrl, Ramp1 and Ramp3, NMU receptors Nmur1 and Nmur2, Vip receptors Vipr1 and Vipr2, Adrb2 (Adrenoceptor beta 2). Vipr2 expression increased significantly following alcohol feeding, whereas Nmur2 expression in ILC2s was downregulated (**Figure 4B**). The changes in the mRNA levels of *Calcr1* and *Ramp1* were less than that in *Vipr2*. Since Vip has an effect on the expression of IL-5 by lung ILC2s in models of allergic airway inflammation, we analyzed the changes in Vip levels after alcohol feeding and found the levels of Vip in serum and lung homogenates were similar in mice fed the alcohol diet and control diet (**Figure 4C**). Thus, to further study the effect of alcohol ingestion on the changes in neuropeptides, we examined the level of CGRP, which is expressed in lung sensory nerves with cell bodies located in the vagal ganglia and in the PNECs. PNECs are specialized epithelial cells that produce neurotransmitters (38). Immunohistochemistry staining showed that CGRP expression remained restricted to PNECs within the lung epithelium and downregulated after alcohol challenge (**Figure 4D**). Furthermore, qRT-PCR analysis showed that *Calca* expression in the vagal ganglia was also reduced in mice fed the alcohol diet compared with mice fed the control diet (**Figure 4E**). Using immunofluorescent, we observed a dense nerve fiber network labeled for the pan-neuronal marker Tuj1 and for CGRP in whole-mount trachea preparations and found the amount of intraepithelial CGRP^+^ nerve fibers was largely reduced after alcohol ingestion (**Figure 4F**). These data indicate that alcohol consumption perturbs the CGRP release, probably from CGRP^+^ nerve and may provide an explanation for the observed ILC2 functional changes in ALI.

### Alcohol-induced lung ILC2s enrichment and neutrophil recruitment are dependent on CGRP

**Figure 5:**
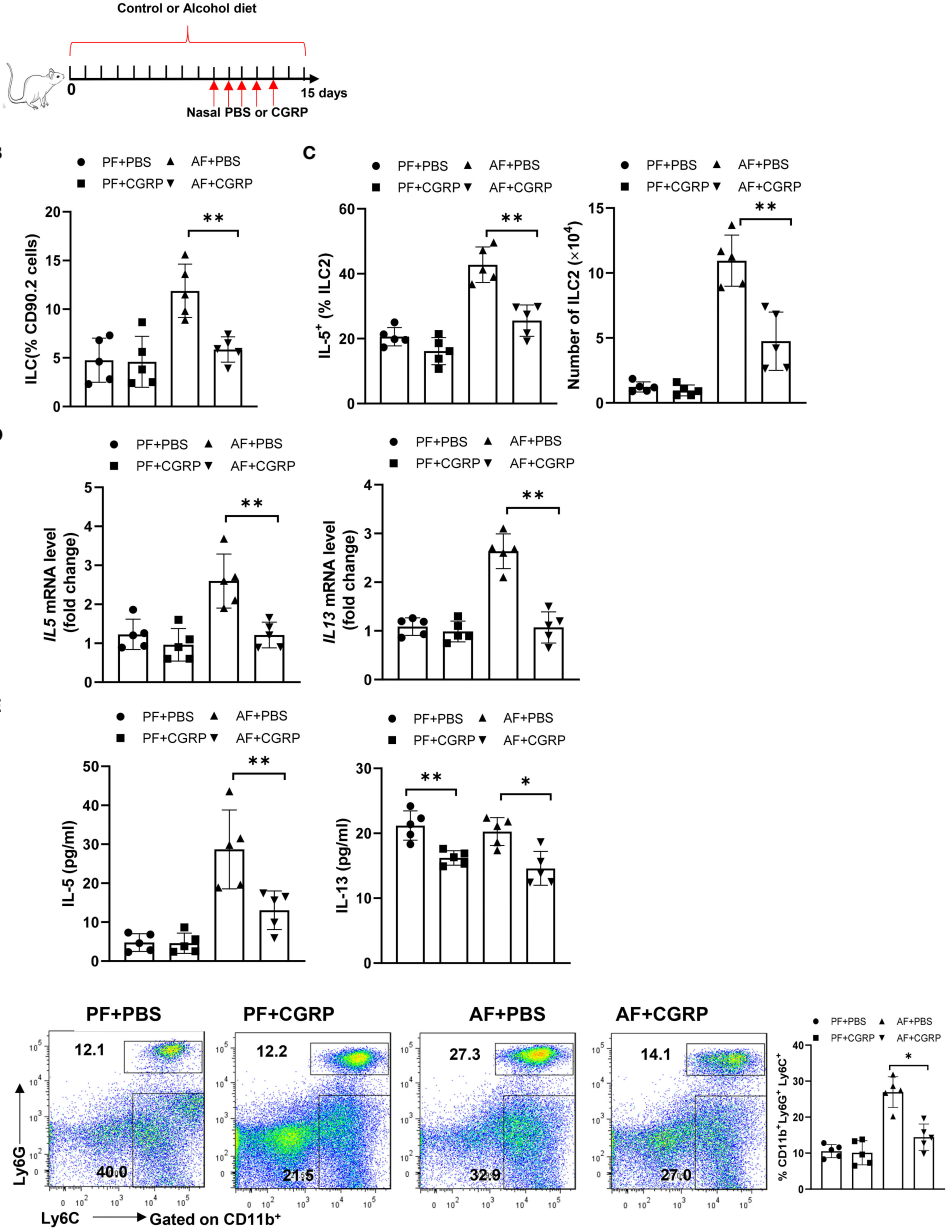
CGRP dampens alcohol-induced type 2 immune response. **(A)** Schematic diagram of nasal administration of CGRP during alcohol feeding. **(B)** Frequency of lung ILCs, determined by flow cytometry. **(C)** Frequency (left) and number (right) of IL-5^+^ ILC2s in whole lung tissues determined by flow cytometry. **(D)** IL-5 and IL-13 expression in lung tissue determined by qRT-PCR. **(E)** IL-5 and IL-13 concentration in lung tissue determined by Multiplex. **(F)** Frequency of lung neutrophils (CD11b^+^Ly6G^+^Ly6C^+^) and monocytes (CD11b^+^Ly6G^-^Ly6C^+^) determined by flow cytometry. Error bars Mean ± SEM; n =5, *p < 0.05, **p < 0.01.

Given that CGRP limits lung-resident ILC2 activation (26) and ILC2s promote pulmonary fibrosis, we evaluated whether administration of CGRP could ameliorate alcohol-driven type 2 inflammation. To test this, we treated control diet- or alcohol diet-fed mice with PBS or CGRP for 5 days and evaluated lung inflammation (**Figure 5A**). The total number or frequency of lung-resident ILCs was not significantly altered by CGRP treatment in PF mice compared with PBS treatment. However, CGRP treatment significantly reduced ILCs numbers and frequencies in AF mice when compared with the PBS treatment in AF mice (**Figure 5B**). As expected, CGRP also reduced the production of type 2 cytokines by ILCs after alcohol feeding, as evidenced by the reduction in the frequency of IL-5^+^ ILCs (**Figure 5C**), lung IL-5 and IL-13 transcripts (**Figure 5D**), and IL-5 and IL-13 proteins in lung homogenates (**Figure 5E**). Furthermore, CGRP administration inhibits alcohol-induced neutrophil recruitment *in vivo* (**Figure 5F**). Together these findings indicate that CGRP is essential for mediating alcohol-induced ILCs enrichment and neutrophil recruitment.

**Figure 6:**
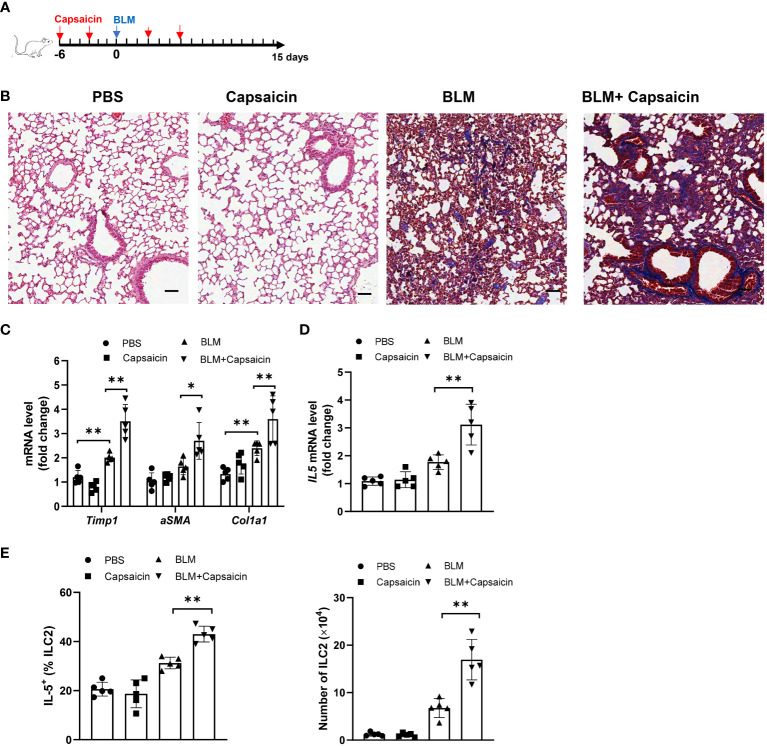
Blockage of CGRP^+^ neuron promotes bleomycin-induced lung injury. Bleomycin-challenged mice were treated with capsaicin (50 mg/kg body weight) or control by subcutaneous injection. **(A)** Schematic diagram of administration of capsaicin during alcohol feeding. BLM, bleomycin. **(B)** Masson’s trichrome staining of lung tissues. Scale bar: 50µM. **(C)** mRNA levels of the fibrotic genes in lung tissues as measured by qRT-PCR. **(D)** IL-5 mRNA level in lung tissues determined by qRT-PCR. **(E)** Frequency (left) of IL-5^+^ ILC2s and number (right) of ILC2s in whole lung tissues determined by flow cytometry. Error bars Mean ± SEM; n =5, *p < 0.05, **p < 0.01, representative of 2 independent experiments **(B)**.

The depletion of CGRP positive nerves is possible by the repeated treatment with TRPV1 agonist, such as resiniferatoxin and capsaicin, resulting in the desensitization of sensory nerves, which is often used as a blocking strategy (39, 40). Given our data that alcohol treatment also suppresses the TRPV1 expression and reduces the number of CGRP^+^ nerves, we next asked whether CGRP release from TRPV1-positive nerve exerts effects on the ILC2 response and ALI. To test this, we depleted endogenous CGRP by injection of capsaicin 6 days before and after bleomycin challenge (**Figure 6A**). Moreover, an increase of collagen deposition was found in the lung of capsaicin-treated mice compared to controls, as evidenced by Masson’s trichrome staining at 15 days after bleomycin challenge (**Figure 6B**). Consistent with the histology data, capsaicin treatment led to an increased levels of lung fibrotic genes, including *αSMA, Col1a1*, and *Timp1* (**Figure 6C**). Intriguingly, capsaicin-treated mice have the marked increases in IL-5 production by lung ILC2s (**Figure 6D**), as well as in the number of ILC2s (**Figure 6E**). Considered together, these results indicate that the production of CGRP from TRPV1^+^ neurons modulates ALI, probably acting through CGRP receptor on ILC2s.

### CGRP-Ramp1 signaling is required for alcohol ingestion-regulated ILC2 responses and lung fibrosis

**Figure 7:**
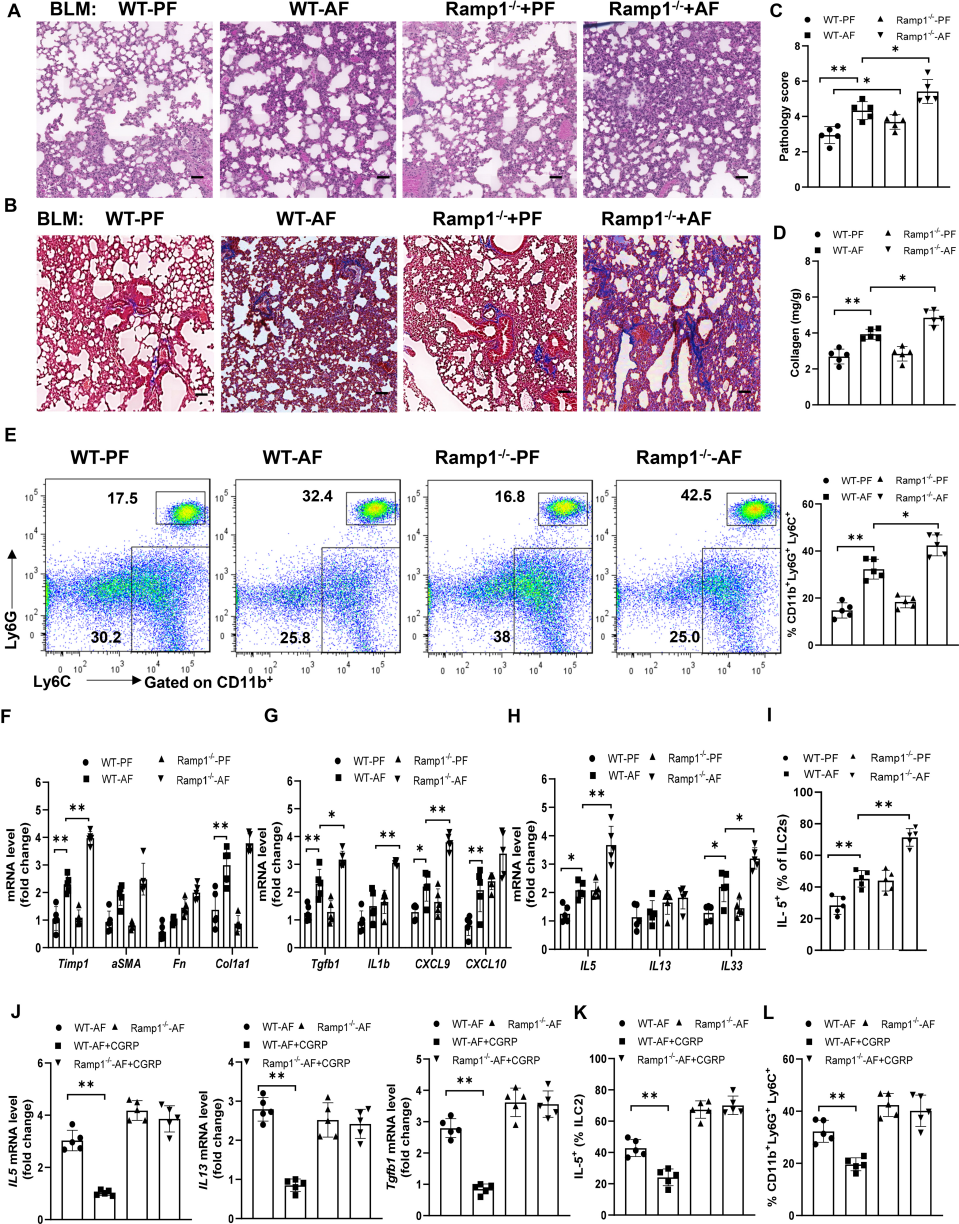
Alcohol ingestion promotes lung type 2 immune response and bleomycin-induced lung injury through CGRP-Ramp1 signaling. **(A-I)** 8-week-old WT mice or Ramp1^-/-^ mice were fed control diet (PF) or alcohol diet (AF) for 14 days and then treated with bleomycin. Lungs were harvested and analyzed at 14 days after bleomycin challenge. **(J-L)** CGRP were administered nasally to WT mice or Ramp1^-/-^ mice without bleomycin treatment during alcohol feeding as **Figure 5**. **(A, B)** Hematoxylin and eosin **(A)** and Masson’s trichrome staining **(B)**. Scale bar: 50µM. **(C)** Lung pathology score. **(D)** Lung tissue collagen content. **(E)** Frequency of lung neutrophils (CD11b^+^Ly6G^+^Ly6C^+^) and monocytes (CD11b^+^Ly6G^-^Ly6C^+^) determined by flow cytometry. **(F-H, J)** mRNA levels of the indicated genes in lung tissues determined by qRT-PCR. **(I, K, L)** Frequency of lung IL-5^+^ ILC2s **(I, K)** and neutrophils **(L)** determined by flow cytometry. Error bars Mean ± SEM; n =5-7, *p < 0.05, **p < 0.01, representative of 3 **(A-I)** or 2 **(J-L)** independent experiments.

To study how endogenous CGRP regulates alcohol-primed lung inflammation, we analyzed wild-type (WT) and *Ramp1* ^-/-^ mice with/without alcohol feeding in bleomycin-induced mice. Although lung fibrosis in Ramp1^-/-^ mice was slightly increased under control diet-fed conditions, fibrosis significantly increased in Ramp1^-/-^ mice under alcohol diet-fed conditions compared with control mice, as evidenced by the staining of H&E and Masson’s trichrome, the pathology score and collagen deposition (**Figures 7A–D**). Flow cytometry analysis showed that a similar number of neutrophils were recruited into the lung of *Ramp1* ^−/−^ mice compared to WT mice on the control diet. However, we observed a large increase in CD11b^+^Ly6G^+^ Ly6C^+^ neutrophils in alcohol-fed *Ramp1* ^-/-^ mice compared to the alcohol-fed control mice (**Figure 7E**). Alcohol treatment led to a similar reduction in CD11b^int^F4/80^-^ myeloid cells in WT and *Ramp1* ^−/−^ mice. By contrast, we did not detect significant differences in CD11b^+^F4/80^hi^ macrophages between two genotypes (**Figure S3**). The expression of fibrosis-associated genes and type 2 inflammatory response-associated genes were also increased in the alcohol-fed *Ramp1* ^-/-^ mice (**Figures 7F–H**). The frequencies of IL-5^+^ ILC2s were also significantly elevated in alcohol-challenged or unchallenged *Ramp1* ^-/-^ mice (**Figure 7I**), indicating that CGRP-ILC2 axis is an important regulator of alcohol-mediated lung injury. Finally, exogenous CGRP failed to inhibit alcohol-induced lung IL-15, IL-13 and TGFβ1 expression, the expansion of neutrophil and ILC2 in Ramp1^-/-^ mice, compared to WT controls (**Figures 7J–L**). To confirm the cell-intrinsic effect of Ramp1 in ILC2s, ILC2s were sorted from WT or Ramp1^-/-^ mice and then transferred intravenously into Rag2^-/-^ Il2rg^-/-^ mice. After 7 days, Alcohol diet were provided and bleomycin was administered intranasally to the recipient mice to induce pulmonary fibrosis. Notably, pulmonary fibrotic responses were greatly enhanced in mice that received Ramp1^-/-^ ILC2s, as evidenced by increased collagen deposition and fibrotic gene expression (**Figures S4A, B**). Consistently, a significant increase of IL-5 production was found in mice Ramp1^-/-^ ILC2s (**Figures S4C**). Thus, these data point to the fact that CGRP-Ramp1 signaling is required for inhibition of ILC2 responses in the mouse alcohol-mediated lung fibrosis model.

## Discussion

Our study reveals a mechanism by which chronic alcohol ingestion exacerbates bleomycin-induced pulmonary fibrosis in mice. Although administration of alcohol on its own was not sufficient to induce lung fibrosis, we found that the profibrogenic effect of alcohol is mainly attributed to its role in neutrophil infiltration and the accumulation of ILC2 into the lung interstitium. An increase in the blood neutrophil-lymphocyte ratio (NLR) is associated with higher susceptibility to infection, acute kidney injury, and poor clinical outcome following steroid treatment in patients with alcohol-associated hepatitis (AH) (41, 42). This might be a consequence of the secretion of the chemokines CXCL9 and CXCL10 (**Figure 1H**). CXCL9 and CXCL10 determine the migration of neutrophils (43), and CCL2 is used for the migration of monocyte/macrophage and lymphocyte into the inflamed lung tissues in mice. Alcoholic hepatitis (AH) is associated with massive neutrophils infiltration in liver (44–46). Our data show that alcohol consumption also induced the infiltration of neutrophil in the lungs. Recent study showed that IL-25 induces concomitant tissue eosinophil and neutrophil infiltration in the lungs in an ILC2 dependent way. It has been reported that ILC2 promote HCC progression through CXCL2‐neutrophil‐induced immunosuppression (47). These findings suggest that the potential contribution of ILC2 to the recruitment of neutrophils in the lungs in our models. On the other hand, neutrophil proteases can process the full-length IL-33 into mature IL-33, which subsequently stimulates ILC2s and macrophages to produce IL-13 (9). IL-13 and mature IL-33 then synergistically increase production of TGF-β1. TGF-β1 is known to promote extracellular matrix production and accumulation along with mesenchymal cell, like fibroblast, proliferation and differentiation (9). Together, alcohol consumption-induced type 2 inflammatory response appears to contribute to the development of lung fibrosis.

Ethanol has rapid acute effects on the function of proteins involved in excitatory and inhibitory synaptic transmission. Ethanol generally potentiates cys-loop ligand-gated ion channels (LGICs) (e.g., GABA _a_ and glycine receptors [GlyRs]) but inhibits ionotropic glutamate receptors (48, 49). We recently demonstrated that chronic alcohol consumption reduces the number of Vip-producing neurons in both the ileal and colonic submucosal plexus, which shapes the α1,2-fucosylation of intestinal epithelial cells (IECs) (23). In our current study, we found that chronic ethanol exposure inhibits the release of the neuropeptide, CGRP, which is expressed by sensory neurons and PNECs, which produce neurotransmitters and soluble mediators. A recent study found that mice with genetic ablation of PNECs have the reduced allergen-induced lung inflammation, which is linked to PNEC expression of CGRP (29). CGRP affects both immune and non-immune cells, particularly has pleiotropic effects on immune responses. CGRP suppresses neutrophil responses and inhibits cytokine production (IL-5 and IL-13) and IL-33-mediated inflammation during lung infection (31). Because these cytokines are mainly produced by ILC2s, this might explain why CGRP administration or ILC2 depletion also reduced neutrophil infiltration and lung inflammation in the setting of bleomycin-induced fibrosis in our study. In addition to classical type 2 cytokines IL-5 and IL-13 produced by lung ILC2, amphiregulin (AREG), a ligand of the epidermal growth factor receptor (EGFR), has recently emerged as a component of the type 2 inflammatory response in the respiratory tract (50, 51). The effect of alcohol ingestion on AREG-expressing ILC2s would also be of interest in the future study.

The neuroimmune interactions at peripheral barrier tissues are increasingly appreciated clinically and experimentally (52, 53). Recent reports showed that neuropeptides directly impact tissue-resident mucosal innate immune cells (54). Specifically, cholinergic neurons adjacent to intestinal and pulmonary ILC2s produce the neuropeptide NMU, which acts via NMUR1 selectively expressed by ILC2s to induce the production of type 2 cytokines (55). Sympathetic neurons also innervate these areas, and their product, β adrenergic agonists counteracts the action of IL-33 and dampen ILC2s responses (56). It was reported that vagal ganglia TRPV1^+^ afferents mediate immunosuppression through release of the neuropeptide CGRP (31). In the present study, we also explored the role of CGRP in the model of bleomycin-induced fibrosis by using capsaicin to deplete CGRP^+^ neurons. Our results imply that alcohol-dependent type 2 immunity is mediated by CGRP because depletion CGRP with capsaicin produced a further increase in lung ILC2s during pulmonary fibrosis.

Finally, our data in the experiments of Ramp1^-/-^ mice and adoptive transfer show that CGRP receptor Ramp1 limits acute lung inflammation by negatively regulating ILC2 responses. The specificity of the requirement for Ramp1 in this process is demonstrated by the fact that alcohol administration causes more lung inflammation in Ramp1*-/-*mice. Thus, alcohol might promote ILC2 activation in bleomycin-induced fibrogenesis via CGRP/Ramp1 signaling. Since antibodies against CGRP (57) or CGRP receptor (58, 59) have been recently approved for clinic treatment of migraines. Our study highlights the importance to monitor the incidence of lung diseases as they enter widespread clinical use, particularly, in treating patients with AUD.

## Materials and methods

### Experimental model and subject details

#### Mice

C57BL/6 mice, Ramp1^-/-^ and Rag1-/- mice were ordered from Jackson Laboratory. Both male and female WT and knockout littermates were used in all of experiments. We used the binge-on-chronic NIAAA (Gao) model with 8-week-old male and female mice. Briefly, male or female mice were acclimated to the Lieber-DeCarli liquid control diet (F1259SP; Bio-Serv, Flemington, NJ) or gradually introduced to and increased on the ethanol diet (5% ethanol-w/v; F1258SP; Bio-Serv) for 5 days. Mice were further feeding with the liquid control (pair feeding, PF) or ethanol diet (alcohol feeding, AF) for an additional 10 (day 15), 17 (day 22), or 23 days (day 28) as our previous report (10). Studies were approved by the University of Louisville Institutional Animal Care and Use Committee.

### Bleomycin-induced lung fibrosis

Mice were fed alcohol or control diet for 15 days (day 15) and then lightly anesthetized with isoflurane gas. PBS control or Bleomycin sulfate (Sigma, B1141000, 2 mg/kg body weight) or was administered intranasally at 30 μl per mouse. Mice were continually on control diet or alcohol diet. Lungs were collected 14 days after bleomycin treatment. Mice received nasally 6.65 μg CGRP for 5 days on day 8 after administration of alcohol. Mice were also given capsaicin (M2028, 50 mg/kg, s.c.; Sigma) one time every three days before and after intratracheal instillation of bleomycin to deplete endogenous CGRP. For ILC depletion, mice were intraperitoneally injected with 200 μg anti-Thy1.2 per mouse (BioXCell) or IgG1 isotype control antibody every three days after administration of bleomycin.

### Adoptive transfer

WT and Ramp1^-/-^ mice were injected with IL-33 (400 ng) of for 4 consecutive days to expand ILC2s. 2 × 10 (5) ILC2s were sorted from WT or Ramp1^-/-^ mice and then transferred intravenously into Rag2^-/-^Il2rg^-/-^ mice. After 7 days, alcohol diet was provided and the recipient mice were administered intranasally with bleomycin to induce pulmonary fibrosis.

### Preparation of lung cells

Lung was cut and digested in 7 ml medium (RPMI 1640 containing collagenase I) (Worthington Biochemical, LS004197) and DNase I (1 mg/ml; Roche, 11284932001) for 45 min at 37°C. Digested tissues were incubated 5 min at 37°C with vigorous shaking. The digested tissues were passed through a 100-μm cell strainer and centrifugated at 2,500 r.p.m. for 20 minutes at room temperature. Mononuclear cells were then collected from the interphase of a 40% and 80% Percoll (GE Healthcare) gradient.

### Histology, immunofluorescence and immunohistochemistry

Tissue specimens were fixed in 10% formalin, dehydrated, and then embedded in paraffin. Tissue samples were cut at the thicknesses of 5 µm. Lung tissues were stained with hematoxylin and eosin and fibrosis was stained using Masson’s trichrome staining (Sigma). In the trichrome-stained sections, the aniline blue-positive areas quantified as a measure of collagen content. Sections were scanned using a PANNORAMIC DESK II DW scanner (3D Histech). For immunofluorescence analysis, OCT-embedded tissue cryosections (6 μm-thick) were blocked for 1 hour at 24°C with DPBS (5% BSA) and incubated overnight at 4°C with the primary antibodies, i.e., Tuj1 [1:500] (Thermo Fisher), rabbit anti-CGRP [1:200] (Sigma), mouse anti-CGRP [1:200] (Abcam). Primary antibodies were detected by Alexa Fluor 488, 594 or 647 conjugated goat anti-rabbit IgG and anti-mouse IgG (1:600, Invitrogen). Tissues were counterstained with DAPI. A Zeiss LSM 510 confocal microscope equipped with a digital image analysis system (Pixera) was used for capturing images. For immunohistochemistry, the slides were first processed for the deparaffinization, rehydration, and antigen retrieval processes as with immunofluorescence staining. The slides were treated with hydrogen peroxide (3%) for 10 min and then blocked with Protein Block Buffer (Vector Laboratories) for 30 min. Sections were stained overnight with anti-MPO or α-SMA (1:100) antibodies, followed by staining with second antibodies (HRP- conjugated anti-IgG). Antigens were then visualized with 3,3′- diaminobenzidine substrate (Vector Laboratories) and scanned using a PANNORAMIC DESK II DW scanner (3D Histech).

### Ashcroft score

Fibrotic scoring was graded by a blinded pathologist according to the scale defined by Ashcroft (60) and modified by Hṻbner et al (61). In each field (20× magnification), the predominant degree of fibrosis was recorded as that occupying more than half of the field area. Scores for each field were added to give a mean of score for each sample of 0-8.

### Reagents, antibodies and flow cytometry

Splenocytes and lung immune cells were incubated with anti-CD16/32 (ThermoFisher Scientific) for 30 min at 4°C, washed with PBS containing 2% FCS, and used for flow cytometry analysis. The following antibodies were used at a dilution of 1/200–1/600: anti-CD4 (RM4-5), anti-CD11b (M1/70), anti-IL-17A (TC11-18H10.1), anti-mouse Ly6G (1A8), anti-IFN-γ (XMG1.2), anti-Foxp3 (FJK-16s), anti-CD3 (145-2C11), anti-Gr-1 (RB6-8C5), CD45.2 (104), anti-CD45 (30-F11), anti-KLRG1 (2F1), anti-CD90.2 (53-2.1), anti-CD127 (A7R34), anti-SCA-1(D7), anti-ST2 (RMST2-33), and anti-IL-5 (TRFK5). All antibodies were ordered from Thermo Fisher unless otherwise noted. For the detection of intracellular cytokines, cells were stimulated with PMA (50 ng/ml) and ionomycin (1 µg/ml) for 1 h and then treated with brefeldin A (5 µg/ml; all obtained from Sigma) for additional 4 h, followed by staining for surface markers. Cells were then fixed, permeabilized, washed and then stained using the Foxp3 Fix/Perm Buffer Set (Thermo Fisher, eBioscience). Flow cytometry data were acquired on a 5-color FACScan (Becton Dickinson) and analyzed using FlowJo software (TreeStar). Cell sorting was performed using a FACSAria II.

### Cytokine analysis

The quantity of IL-13, ST-2, IL-33, IL-5, and IFN-γ was determined in serum and lung tissue using ELISA kits (all from eBioscience, Thermo Fisher). The sensitivity of the assays was <5 pg/ml. Mouse lung samples were also evaluated for cytokine levels using the U-PLEX mouse cytokine 19-plex kit from Meso Scale Discovery (MSD, Cat. No. K15069M-1). Capture antibodies were preincubated in the MSD multiplex assay plates. Standards or samples were pre-diluted with assay diluent and added to plates (50 μl/well) and incubated at room temperature with agitation. The plates were washed after a two-hour incubation. Sulfo-tagged detection antibodies were then added for two hours at room temperature. Plates were washed once again after the incubation. 2X Read Substrate was added. Plates were read on MSD reader and data were analyzed by MSD Discovery Workbench^®^ Software 4.0.

### RNA extraction and PCR

RNA isolation, cDNA reverse transcription, quantitative real-time PCR, and relative quantification using the 2^–ΔCT^ method were carried out as previously reported by our lab. Total RNA was isolated from the lung tissue using the Qiagen RNeasy RNA Isolation Kit. RNA (1μg) was reverse-transcribed with Superscript IV and random primers (Thermo Fisher) for synthesizing cDNA. cDNA samples were then amplified using the applied biosystems Realtime System with SYBR Green Master Mix (Thermo Fisher) and specific primers (**Table S1**) to detect the genes of interest. the δCT method was used for determining fold changes in mRNA expression between treatments and controls. All primers were purchased from Eurofins MWG Operon or Sigma. Results were normalized to the concentration of β-actin mRNA or GAPDH mRNA measured in the same samples and expressed as fold increase over baseline levels that were set at a value of 1. A two-sided Student’s *t*-test and one-way ANOVA were used for determining the differences between groups. Error bars on plots represent ± SEM, unless otherwise noted.

### Quantification and statistical analysis

Student’s *t* tests were used and values are shown as Mean ± SEM. n means the number of independent samples. The asterisks indicate significant differences (p < 0.05). * indicates p < 0.05, **p < 0.01.

## Data availability statement

The original contributions presented in the study are included in the article/**Supplementary Material**. Further inquiries can be directed to the corresponding author.

## Ethics statement

The animal study was reviewed and approved by University of Louisville IACUC.

## Author contributions

LC, RS, CL, and ZX performed the experiments and interpret the data. YS interpreted the findings. LC and ZD designed the study, analyzed and interpreted the data, and prepared the manuscript. All authors contributed to the article and approved the submitted version.

## Supplementary material

Supplementary Figure 1 Chronic alcohol ingestion promotes bleomycin-induced lung injury. 8-week-old mice were fed control diet (PF) or alcohol diet (AF) for 14 days and then treated with bleomycin. Lungs were harvested and analyzed at 14 days after bleomycin challenge. **(A)** Immunohistochemistry staining of myeloperoxidase (MPO) in the lung (arrows indicate MPO positive cells). **(B)** Flow cytometry analysis of lung CD11b^+^F4/80^+^ macrophages and CD11^int^F4/80^-^ myeloid cells. Error bars Mean ± SEM; n =5, *p < 0.05, **p < 0.01. Scale bar: 50µM.

Supplementary Figure 2 Effect of alcohol feeding on the ratio of Th1, Th2, Th17 and Treg cells in lung. 8-week-old mice were fed control diet (PF) or alcohol diet (AF) for 14 days and then treated with bleomycin. Mice were continually on control diet or alcohol diet. Lungs were harvested and analyzed at 14 days after bleomycin or PBS challenge., **(A, B)** Flow cytometry analysis of lung Th2 cells. **(C)** Flow cytometry analysis of lung Th1 cells, Th17 cells and Tregs. **(D)** Soluble ST2, IL-33 and IL-13 concentration in lung tissue determined by ELISA. Error bars Mean ± SEM; n =5, **p < 0.01. ns: no significant.

Supplementary Figure 3 Alcohol ingestion promotes lung type 2 immune response and bleomycin-induced lung injury through CGRP-Ramp1 signaling. 8-week-old WT mice or Ramp1^-/-^ mice were fed control diet (PF) or alcohol diet (AF) and then treated with bleomycin. Lungs were harvested at 14 days. Flow cytometry analysis of lung CD11b^+^F4/80^+^ macrophages and CD11^int^F4/80^-^ myeloid cells. Error bars Mean ± SEM; n =5, **p < 0.01.

Supplementary Figure 4 CGRP- Ramp1 ILC2 axis regulates acute lung injury in a cell-intrinsic manner in alcohol-primed and bleomycin-treated mice. 2 × 10 (5) ILC2s were sorted from WT or Ramp1^-/-^ mice and then transferred intravenously into Rag2^-/-^ Il2rg^-/-^ mice. After 7 days, alcohol diet was provided and bleomycin was administered intranasally to the recipient mice to induce pulmonary fibrosis. **(A)** Masson’s trichrome staining. **(B)** mRNA levels of the indicated genes related to fibrosis in lung. Fn: Fibronectin. **(C, D)** The frequency **(C)** and number **(D)** of IL-5^+^ ILC2s in whole lung tissues, determined by flow cytometry. Error bars Mean ± SEM; n =4, *p < 0.05, **p < 0.01.

## References

[1] Avila MA Dufour JF Gerbes AL Zoulim F Bataller R Burra P. Recent advances in alcohol-related liver disease (ALD): summary of a gut round table meeting. Gut (2020) 69:764–80. doi: 10.1136/gutjnl-2019-319720 PMC7236084 31879281

[2] Gao B Ahmad MF Nagy LE Tsukamoto H. Inflammatory pathways in alcoholic steatohepatitis. J Hepatol (2019) 70:249–59. doi: 10.1016/j.jhep.2018.10.023 PMC6361545 30658726

[3] Mirijello A Tarli C Vassallo GA Sestito L Antonelli M d’Angelo C. Alcoholic cardiomyopathy: what is known and what is not known. Eur J Intern Med (2017) 43:1–5. doi: 10.1016/j.ejim.2017.06.014 28647343

[4] Smith IF Gursky ZH Klintsova AY. Representation of prefrontal axonal efferents in the thalamic nucleus reuniens in a rodent model of fetal alcohol exposure during third trimester. Front Behav Neurosci (2022) 16:993601. doi: 10.3389/fnbeh.2022.993601 36160686 PMC9493097

[5] Sueblinvong V Kerchberger VE Saghafi R Mills ST Fan X Guidot DM. Chronic alcohol ingestion primes the lung for bleomycin-induced fibrosis in mice. Alcoholism-Clinical Exp Res (2014) 38:336–43. doi: 10.1111/acer.12232 PMC3869877 24033682

[6] Simet SM Wyatt TA DeVasure J Yanov D Allen-Gipson D Sisson JH. Alcohol increases the permeability of airway epithelial tight junctions in beas-2B and NHBE cells. Alcoholism-Clinical Exp Res (2012) 36:432–42. doi: 10.1111/j.1530-0277.2011.01640.x PMC3251733 21950588

[7] Guidot DM Hart CM. Alcohol abuse and acute lung injury: epidemiology and pathophysiology of a recently recognized association. J Invest Med (2005) 53:235–45. doi: 10.2310/6650.2005.53506 16042957

[8] Mouratis MA Aidinis V. Modeling pulmonary fibrosis with bleomycin. Curr Opin Pulm Med (2011) 17:355–61. doi: 10.1097/MCP.0b013e328349ac2b 21832918

[9] Li D Guabiraba R Besnard AG Komai-Koma M Jabir MS Zhang L. IL-33 promotes ST2-dependent lung fibrosis by the induction of alternatively activated macrophages and innate lymphoid cells in mice. J Allergy Clin Immunol (2014) 134:1422–1432 e11. doi: 10.1016/j.jaci.2014.05.011 24985397 PMC4258609

[10] Chu S Sun R Gu X Chen L Liu M Guo H. Inhibition of sphingosine-1-Phosphate-Induced Th17 cells ameliorates alcohol-associated steatohepatitis in mice. Hepatology (2021) 73:952–67. doi: 10.1002/hep.31321 PMC8009334 32418220

[11] Artis D Spits H. The biology of innate lymphoid cells. Nature (2015) 517:293–301. doi: 10.1038/nature14189 25592534

[12] Kotas ME Locksley RM. Why innate lymphoid cells? Immunity (2018) 48:1081–90. doi: 10.1016/j.immuni.2018.06.002 PMC6145487 29924974

[13] Maazi H Patel N Sankaranarayanan I Suzuki Y Rigas D Soroosh P. ICOS : ICOS-ligand interaction is required for type 2 innate lymphoid cell function, homeostasis, and induction of airway hyperreactivity. Immunity (2015) 42:538–51. doi: 10.1016/j.immuni.2015.02.007 PMC4366271 25769613

[14] McHedlidze T Waldner M Zopf S Walker J Rankin AL Schuchmann M. Interleukin-33-dependent innate lymphoid cells mediate hepatic fibrosis. Immunity (2013) 39:357–71. doi: 10.1016/j.immuni.2013.07.018 PMC4172965 23954132

[15] Liew FY Girard JP Turnquist HR. Interleukin-33 in health and disease. Nat Rev Immunol (2016) 16:676–89. doi: 10.1038/nri.2016.95 27640624

[16] Wallrapp A Riesenfeld SJ Burkett PR Kuchroo VK. Type 2 innate lymphoid cells in the induction and resolution of tissue inflammation. Immunol Rev (2018) 286:53–73. doi: 10.1111/imr.12702 30294962 PMC7370855

[17] Duerr CU McCarthy CDA Mindt BC Rubio M Meli AP Pothlichet J. Type I interferon restricts type 2 immunopathology through the regulation of group 2 innate lymphoid cells. Nat Immunol (2016) 17:65. doi: 10.1038/ni.3308 26595887 PMC9135352

[18] Moro K Kabata H Tanabe M Koga S Takeno N Mochizuki M. Interferon and IL-27 antagonize the function of group 2 innate lymphoid cells and type 2 innate immune responses. Nat Immunol (2016) 17:76. doi: 10.1038/ni.3309 26595888

[19] Taylor S Huang YF Mallett G Stathopoulou C Felizardo TC Sun MA. PD-1 regulates KLRG1(+) group 2 innate lymphoid cells. J Exp Med (2017) 214:1663–78. doi: 10.1084/jem.20161653 PMC5461001 28490441

[20] Barnig C Cernadas M Dutile S Liu X Perrella MA Kazani S. Lipoxin A4 regulates natural killer cell and type 2 innate lymphoid cell activation in asthma. Sci Transl Med (2013) 5:174ra26. doi: 10.1126/scitranslmed.3004812 PMC3823369 23447017

[21] von Moltke J O’Leary CE Barrett NA Kanaoka Y Austen KF Locksley RM. Leukotrienes provide an NFAT-dependent signal that synergizes with IL-33 to activate ILC2s. J Exp Med (2017) 214:27–37. doi: 10.1084/jem.20161274 28011865 PMC5206504

[22] Rose AK Shaw SG Prendergast MA Little HJ. The importance of glucocorticoids in alcohol dependence and neurotoxicity. Alcoholism-Clinical Exp Res (2010) 34:2011–8. doi: 10.1111/j.1530-0277.2010.01298.x PMC3058879 21087289

[23] Lei C Sun R Xu GZ Tan Y Feng WK McClain CJ. Enteric VIP-producing neurons maintain gut microbiota homeostasis through regulating epithelium fucosylation. Cell Host Microbe (2022) 30:1417. doi: 10.1016/j.chom.2022.09.001 36150396 PMC9588764

[24] Cardoso V Chesne J Ribeiro H Garcia-Cassani B Carvalho T Bouchery T. Neuronal regulation of type 2 innate lymphoid cells via neuromedin U. Nature (2017) 549:277–81. doi: 10.1038/nature23469 PMC5714273 28869974

[25] Wallrapp A Riesenfeld SJ Burkett PR Abdulnour RE Nyman J Dionne D. The neuropeptide NMU amplifies ILC2-driven allergic lung inflammation. Nature (2017) 549:351–6. doi: 10.1038/nature24029 PMC5746044 28902842

[26] Nagashima H Mahlakoiv T Shih HY Davis FP Meylan F Huang Y. Neuropeptide CGRP limits group 2 innate lymphoid cell responses and constrains type 2 inflammation. Immunity (2019) 51:682–695 e6. doi: 10.1016/j.immuni.2019.06.009 31353223 PMC6801073

[27] Holzmann B. Modulation of immune responses by the neuropeptide CGRP. Amino Acids (2013) 45:1–7. doi: 10.1007/s00726-011-1161-2 22113645

[28] Cutz E Pan J Yeger H Domnik NJ Fisher JT. Recent advances and contraversies on the role of pulmonary neuroepithelial bodies as airway sensors. Semin Cell Dev Biol (2013) 24:40–50. doi: 10.1016/j.semcdb.2012.09.003 23022441

[29] Branchfield K Nantie L Verheyden JM Sui PF Wienhold MD Sun X. Pulmonary neuroendocrine cells function as airway sensors to control lung immune response. Science (2016) 351:707–10. doi: 10.1126/science.aad7969 PMC4860346 26743624

[30] Xu HP Ding JR Porter CBM Wallrapp A Tabaka M Ma S. Transcriptional atlas of intestinal immune cells reveals that neuropeptide alpha-CGRP modulates group 2 innate lymphoid cell responses. Immunity (2019) 51:696. doi: 10.1016/j.immuni.2019.09.004 31618654 PMC6991097

[31] Baral P Umans BD Li L Wallrapp A Bist M Kirschbaum T. Nociceptor sensory neurons suppress neutrophil and gammadelta T cell responses in bacterial lung infections and lethal pneumonia. Nat Med (2018) 24:417–26. doi: 10.1038/nm.4501 PMC6263165 29505031

[32] Ammendola A Gemini D Iannaccone S Argenzio F Ciccone G Ammendola E. Gender and peripheral neuropathy in chronic alcoholism: a clinical-electroneurographic study. Alcohol Alcoholism (2000) 35:368–71. doi: 10.1093/alcalc/35.4.368 10906002

[33] Chopra K Tiwari V. Alcoholic neuropathy: possible mechanisms and future treatment possibilities. Brit J Clin Pharmaco (2012) 73:348–62. doi: 10.1111/j.1365-2125.2011.04111.x PMC3370340 21988193

[34] Li S Koziol-White C Jude J Jiang M Zhao H Cao G. Epithelium-generated neuropeptide y induces smooth muscle contraction to promote airway hyperresponsiveness. J Clin Invest (2016) 126:1978–82. doi: 10.1172/JCI81389 PMC4855915 27088802

[35] Nussbaum JC Van Dyken SJ von Moltke J Cheng LE Mohapatra A Molofsky AB. Type 2 innate lymphoid cells control eosinophil homeostasis. Nature (2013) 502:245–8. doi: 10.1038/nature12526 PMC3795960 24037376

[36] Meseguer V Alpizar YA Luis E Tajada S Denlinger B Fajardo O. TRPA1 channels mediate acute neurogenic inflammation and pain produced by bacterial endotoxins. Nat Commun (2014) 5:3125. doi: 10.1038/ncomms4125 24445575 PMC3905718

[37] Hollenhorst MI Nandigama R Evers SB Gamayun I Abdel Wadood N Salah A. Bitter taste signaling in tracheal epithelial brush cells elicits innate immune responses to bacterial infection. J Clin Invest (2022) 132(13):e150951. doi: 10.1172/JCI150951 35503420 PMC9246383

[38] Domnik NJ Cutz E. Pulmonary neuroepithelial bodies as airway sensors: putative role in the generation of dyspnea. Curr Opin Pharmacol (2011) 11:211–7. doi: 10.1016/j.coph.2011.04.003 21530400

[39] Banvolgyi A Palinkas L Berki T Clark N Grant AD Helyes Z. Evidence for a novel protective role of the vanilloid TRPV1 receptor in a cutaneous contact allergic dermatitis model. J Neuroimmunol (2005) 169:86–96. doi: 10.1016/j.jneuroim.2005.08.012 16188326

[40] Kee Z Kodji X Brain SD. The role of calcitonin gene related peptide (CGRP) in neurogenic vasodilation and its cardioprotective effects. Front Physiol (2018) 9. doi: 10.3389/fphys.2018.01249 PMC6156372 30283343

[41] Cho Y Szabo G. Two faces of neutrophils in liver disease development and progression. Hepatology (2021) 74:503–12. doi: 10.1002/hep.31680 PMC9235297 33314193

[42] Das S Maras JS Hussain MS Sharma S David P Sukriti S. Hyperoxidized albumin modulates neutrophils to induce oxidative stress and inflammation in severe alcoholic hepatitis. Hepatology (2017) 65:631–46. doi: 10.1002/hep.28897 27775820

[43] Metzemaekers M Gouwy M Proost P. Neutrophil chemoattractant receptors in health and disease: double-edged swords. Cell Mol Immunol (2020) 17:433–50. doi: 10.1038/s41423-020-0412-0 PMC7192912 32238918

[44] Ma J Guillot A Yang Z Mackowiak B Hwang S Park O. Distinct histopathological phenotypes of severe alcoholic hepatitis suggest different mechanisms driving liver injury and failure. J Clin Invest (2022) 132(14):e157780. doi: 10.1172/JCI157780 35838051 PMC9282929

[45] Szabo G Saha B. Alcohol’s effect on host defense. Alcohol Res (2015) 37:159–70. 10.35946/arcr.v37.2.01 PMC4590613 26695755

[46] Cho Y Bukong TN Tornai D Babuta M Vlachos IS Kanata E. Neutrophil extracellular traps contribute to liver damage and increase defective low-density neutrophils in alcohol-associated hepatitis. J Hepatol (2023) 78:28–44. doi: 10.1016/j.jhep.2022.08.029 36063965 PMC11910133

[47] Xu XY Ye LY Zhang Q Shen H Li SS Zhang XY. Group-2 innate lymphoid cells promote HCC progression through CXCL2-Neutrophil-Induced immunosuppression. Hepatology (2021) 74:2526–43. doi: 10.1002/hep.31855 PMC8597094 33829508

[48] Lovinger DM Roberto M. Synaptic effects induced by alcohol. Curr Top Behav Neurosci (2013) 13:31–86. doi: 10.1007/978-3-642-28720-6_143 21786203 PMC4791588

[49] Soderpalm B Lido HH Ericson M. The glycine receptor-a functionally important primary brain target of ethanol. Alcohol Clin Exp Res (2017) 41:1816–30. doi: 10.1111/acer.13483 28833225

[50] Monticelli LA Sonnenberg GF Abt MC Alenghat T Ziegler CG Doering TA. Innate lymphoid cells promote lung-tissue homeostasis after infection with influenza virus. Nat Immunol (2011) 12:1045–54. doi: 10.1038/ni.2131 PMC3320042 21946417

[51] Starkey MR McKenzie AN Belz GT Hansbro PM. Pulmonary group 2 innate lymphoid cells: surprises and challenges. Mucosal Immunol (2019) 12:299–311. doi: 10.1038/s41385-018-0130-4 30664706 PMC6436699

[52] Klose CS Artis D. Neuronal regulation of innate lymphoid cells. Curr Opin Immunol (2019) 56:94–9. doi: 10.1016/j.coi.2018.11.002 30530300

[53] Veiga-Fernandes H Mucida D. Neuro-immune interactions at barrier surfaces. Cell (2016) 165:801–11. doi: 10.1016/j.cell.2016.04.041 PMC4871617 27153494

[54] Veiga-Fernandes H Artis D. Neuronal-immune system cross-talk in homeostasis interactions between immune and neuronal cells are pillars in tissue homeostasis. Science (2018) 359:1465–6. doi: 10.1126/science.aap9598 29599230

[55] Klose CSN Mahlakoiv T Moeller JB Rankin LC Flamar AL Kabata H. The neuropeptide neuromedin U stimulates innate lymphoid cells and type 2 inflammation. Nature (2017) 549:282. doi: 10.1038/nature23676 28869965 PMC6066372

[56] Moriyama S Brestoff JR Flamar AL Moeller JB Klose CSN Rankin LC. beta(2)-adrenergic receptor-mediated negative regulation of group 2 innate lymphoid cell responses. Science (2018) 359:1056–61. doi: 10.1126/science.aan4829 29496881

[57] Edvinsson L. CGRP antibodies as prophylaxis in migraine. Cell (2018) 175:1719–9. doi: 10.1016/j.cell.2018.11.049 30550780

[58] Goadsby PJ Reuter U Hallstrom Y Broessner G Bonner JH Zhang F. A controlled trial of erenumab for episodic migraine. New Engl J Med (2017) 377:2123–32. doi: 10.1056/NEJMoa1705848 29171821

[59] Silberstein SD Dodick DW Bigal ME Yeung PP Goadsby PJ Blankenbiller T. Fremanezumab for the preventive treatment of chronic migraine. N Engl J Med (2017) 377:2113–22. doi: 10.1056/NEJMoa1709038 29171818

[60] Ashcroft T Simpson JM Timbrell V. Simple method of estimating severity of pulmonary fibrosis on a numerical scale. J Clin Pathol (1988) 41:467–70. doi: 10.1136/jcp.41.4.467 PMC1141479 3366935

[61] Hubner RH Gitter W El Mokhtari NE Mathiak M Both M Bolte H. Standardized quantification of pulmonary fibrosis in histological samples. Biotechniques (2008) 44:507–11, 514-7. doi: 10.2144/000112729 18476815

