# Supplementary material for: Alcohol-mediated susceptibility to lung fibrosis is associated with group 2 innate lymphoid cells in mice
Source: Front Immunol. 2023 Jun 29;14:1178498. doi: 10.3389/fimmu.2023.1178498 (PMC10343460; doi:10.3389/fimmu.2023.1178498)
Supplement: Supplementary file 1 [file Table_1.docx]

**Table S1. Primers used for Real-time PCR**

| Gene name | Forward primer | Reverse primer |
| --- | --- | --- |
| *GAPDH* | AGGTCATCCCAGAGCTGAACG | ACCCTGTTGCTGTAGCCGTAT |
| *β-Actin* | ACGGCCAGGTCATCACTATTC | AGGAAGGCTGGAAAAGAGCC |
| *IL-1β* | GCAACTGTTCCTGAACTCAACT | ATCTTTTGGGGTCCGTCAACT |
| *Calca* | CCTTTCCTGGTTGTCAGCATCTTG | CTGGGCTGCTTTCCAAGATTGAC |
| Calcrl | ACTGTGGTGTCATTCTCAGCC | ATTGGCACTTCAGCTTTAGAGA |
| Npy | TGGCCAGATACTACTCCGCT | TTGTTCTGGGGGCGTTTTCT |
| *Vip* | GGCTTTGCAATCCCCAAAGG | ACACATCCATAGCACACGCA |
| Ramp1 | CACTGAGAAATCCGGCCCAT | CAGTCACACCACAGCGTCTC |
| *Nmur1* | TCAAGTGGCCCTATCAACCTG | CAGATGGGGACAAACTGTTTCAT |
| *Nmur2* | CAAGCGCAGTGACCTATCCC | GGCATACCTAAGAGCAGGACC |
| Ramp3 | GTGAGTGTGCCCAGGTATGC | CGACAGGTTGCACCACTTC |
| *Vipr1* | GATGTGGGACAACCTCACCTG | TAGCCGTGAATGGGGGAAAAC |
| *Vipr2* | GACCTGCTACTGCTGGTTG | CAGCTCTGCACATTTTGTCTCT |
| *Chga* | TTCCCACTTCCATGCAGGCTAC | GCCTCTGTCTTTCCATCTCCATCC |
| *TRPV1* | CCGGCTTTTTGGGAAGGGT | GAGACAGGTAGGTCCATCCAC |
| *ST2* | TCTGTGGAGTACTTTGTTCACC | TCTGCTATTCTGGATACTGCTTTC |
| *IL-33* | TCCAACTCCAAGATTTCCCCG | CATGCAGTAGACATGGCAGAA |
| *IL-5* | CTCTGTTGACAAGCAATGAGACG | TCTTCAGTATGTCTAGCCCCTG |
| *IL-13* | CCTGGCTCTTGCTTGCCTT | GGTCTTGTGTGATGTTGCTCA |
| *CXCL9* | TCCTTTTGGGCATCATCTTCC | TTTGTAGTGGATCGTGCCTCG |
| *CXCL10* | CCAAGTGCTGCCGTCATTTTC | GGCTCGCAGGGATGATTTCAA |
| *Col1a1* | CCCGCCGATGTCGCTAT | GCTACGCTGTTCTTGCAGTGAT |
| *α-SMA* | GTGAAGAGGAAGACAGCACAG | GCCCATTCCAACCATTACTCC |
| *TIMP 1* | CACAAGCCTGGATTCCGTGG | TCCCTTGCAAACTGGAGAGTGAC |
| *Fibronectin* | GGTCTGCAGAGGTTGACAGTG | GGAGAAGTTTGTGCATGGTGTCC |
| *Nmu-F* | GAGGGAGCTTTGCCGTATAGT | GATGCACAACAGAGGACACAA |
| *Adrb2-F* | GGGAACGACAGCGACTTCTT | GCCAGGACGATAACCGACAT |
| *Dbh-F* | GAGGCGGCTTCCATGTACG | TCCAGGGGGATGTGGTAGG |
