# Supplementary figures and images for: Alcohol-mediated susceptibility to lung fibrosis is associated with group 2 innate lymphoid cells in mice
Source: Front Immunol. 2023 Jun 29;14:1178498. doi: 10.3389/fimmu.2023.1178498 (PMC10343460; doi:10.3389/fimmu.2023.1178498)

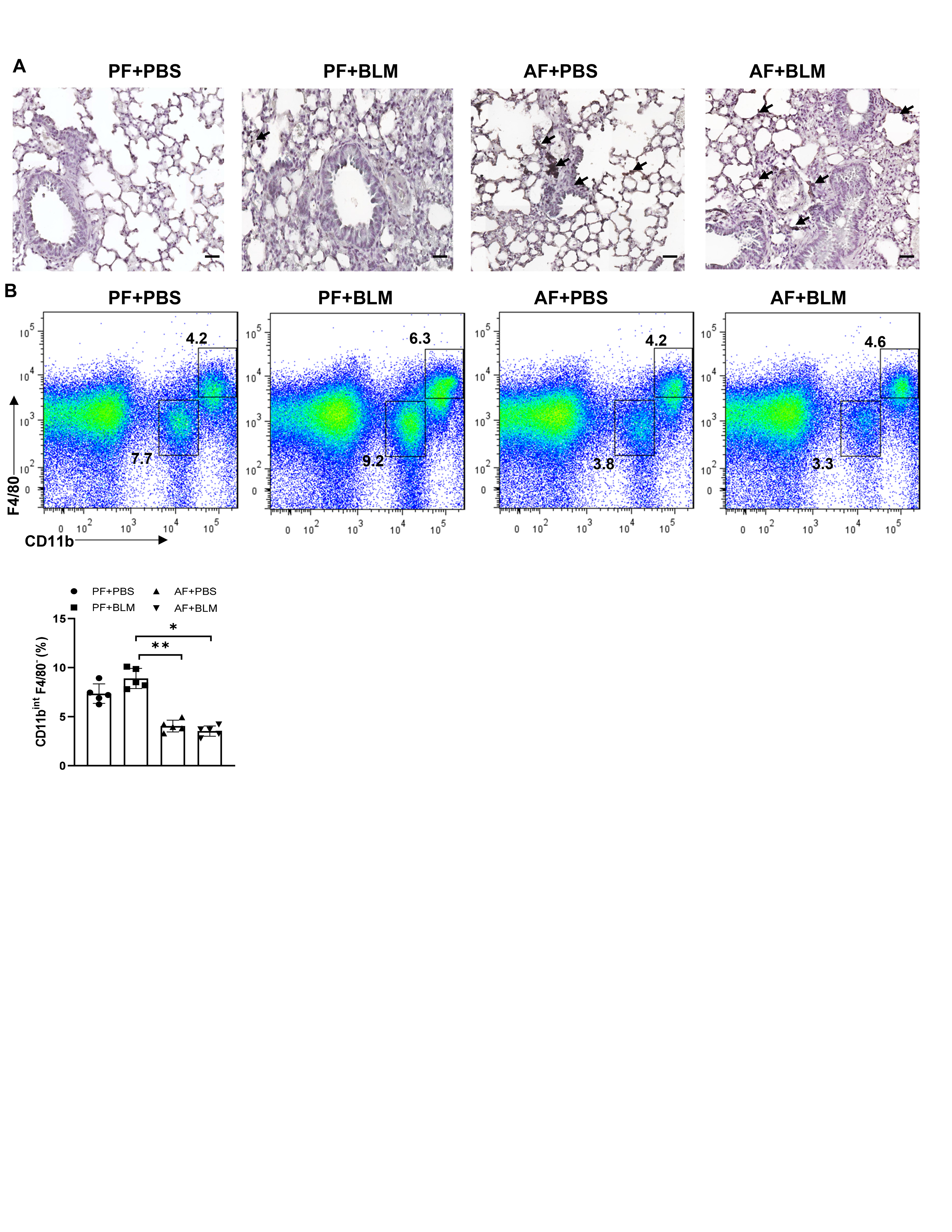

Supplement: Supplementary Figure 1 — Chronic alcohol ingestion promotes bleomycin-induced lung injury. 8-week-old mice were fed control diet (PF) or alcohol diet (AF) for 14 days and then treated with bleomycin. Lungs were harvested and analyzed at 14 days after bleomycin challenge. (A) Immunohistochemistry staining of myeloperoxidase (MPO) in the lung (arrows indicate MPO positive cells). (B) Flow cytometry analysis of lung CD11b+F4/80+ macrophages and CD11intF4/80- myeloid cells. Error bars Mean ± SEM; n =5, *p < 0.05, **p < 0.01. Scale bar: 50µM. [file Image_1.jpeg]

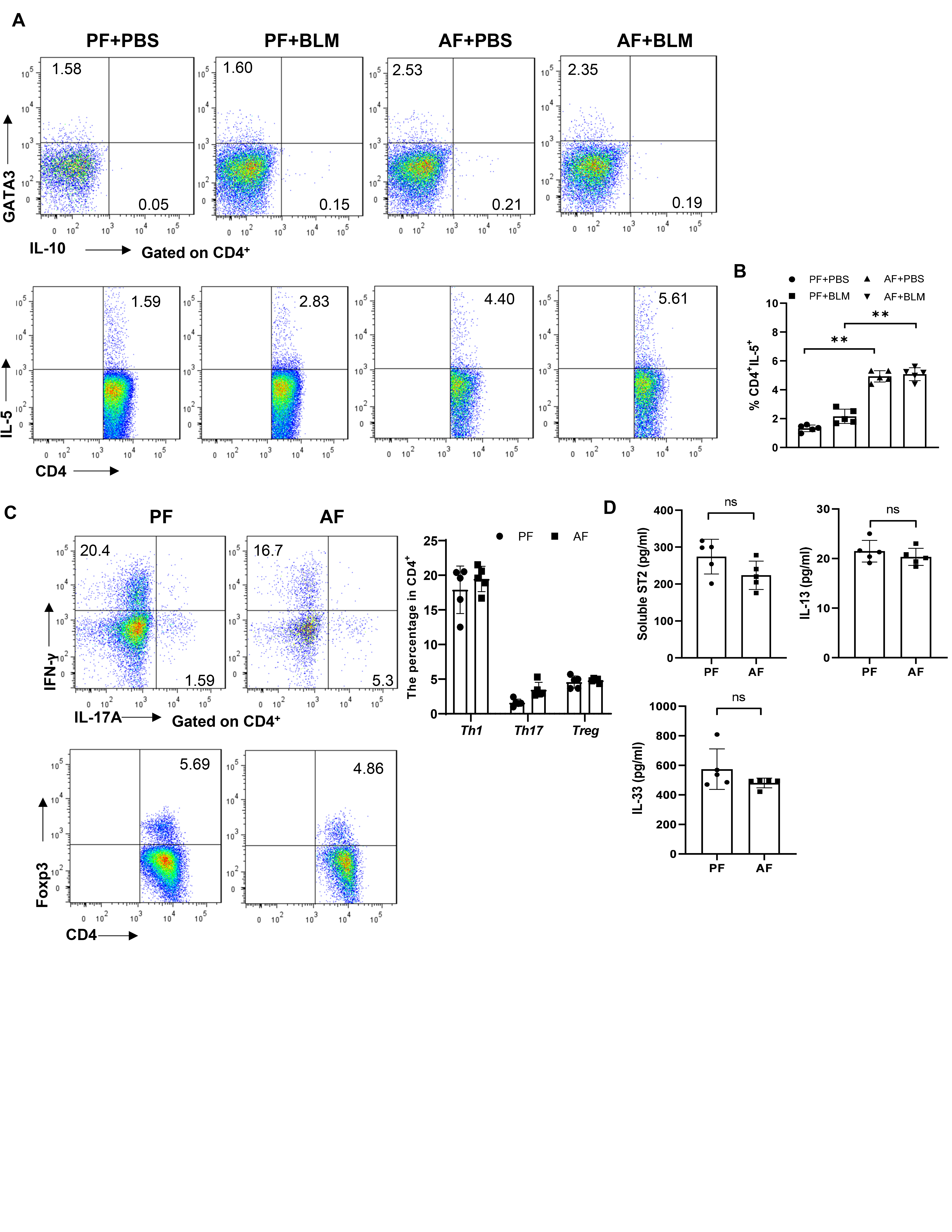

Supplement: Supplementary Figure 2 — Effect of alcohol feeding on the ratio of Th1, Th2, Th17 and Treg cells in lung. 8-week-old mice were fed control diet (PF) or alcohol diet (AF) for 14 days and then treated with bleomycin. Mice were continually on control diet or alcohol diet. Lungs were harvested and analyzed at 14 days after bleomycin or PBS challenge., (A, B) Flow cytometry analysis of lung Th2 cells. (C) Flow cytometry analysis of lung Th1 cells, Th17 cells and Tregs. (D) Soluble ST2, IL-33 and IL-13 concentration in lung tissue determined by ELISA. Error bars Mean ± SEM; n =5, **p < 0.01. ns: no significant. [file Image_2.jpeg]

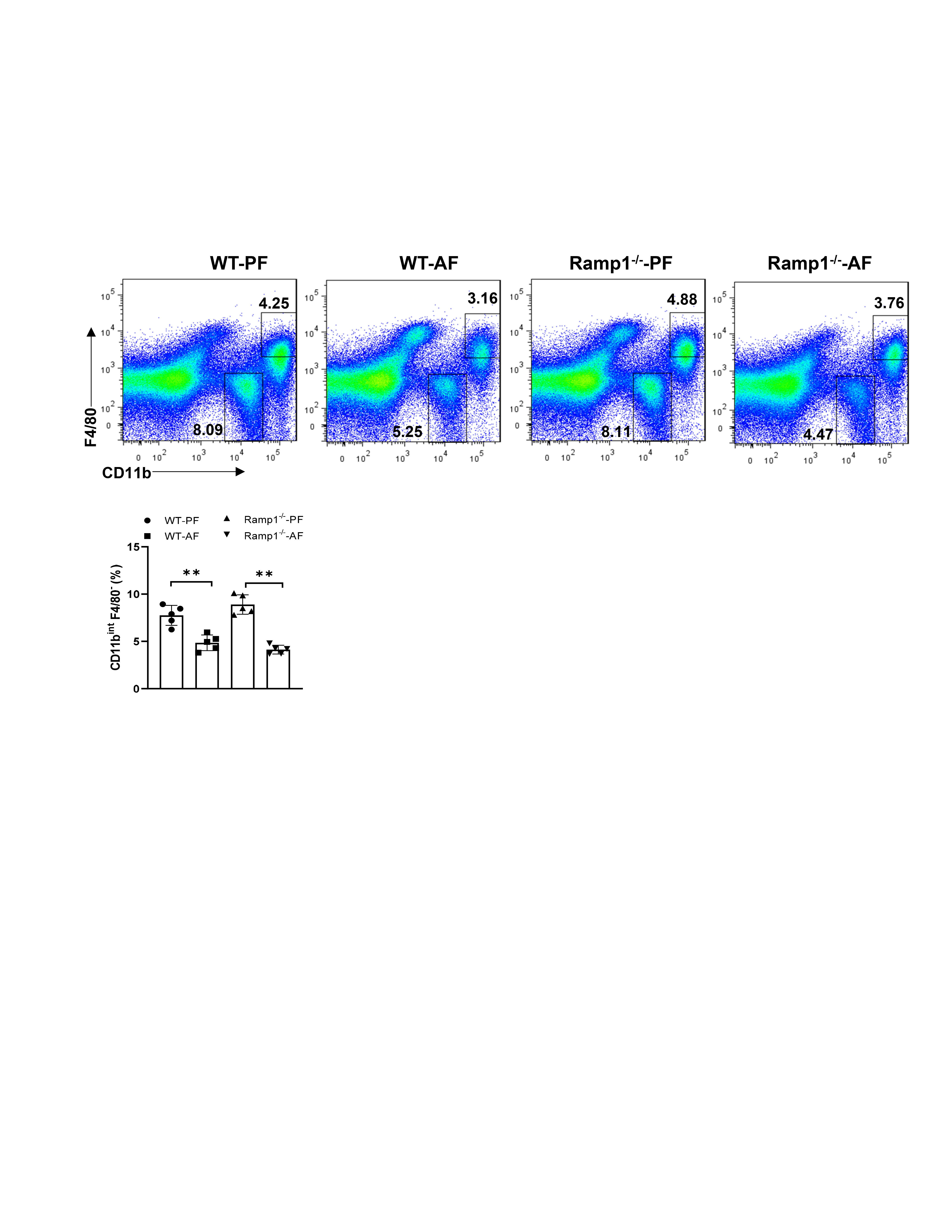

Supplement: Supplementary Figure 3 — Alcohol ingestion promotes lung type 2 immune response and bleomycin-induced lung injury through CGRP-Ramp1 signaling. 8-week-old WT mice or Ramp1-/- mice were fed control diet (PF) or alcohol diet (AF) and then treated with bleomycin. Lungs were harvested at 14 days. Flow cytometry analysis of lung CD11b+F4/80+ macrophages and CD11intF4/80- myeloid cells. Error bars Mean ± SEM; n =5, **p < 0.01. [file Image_3.jpeg]

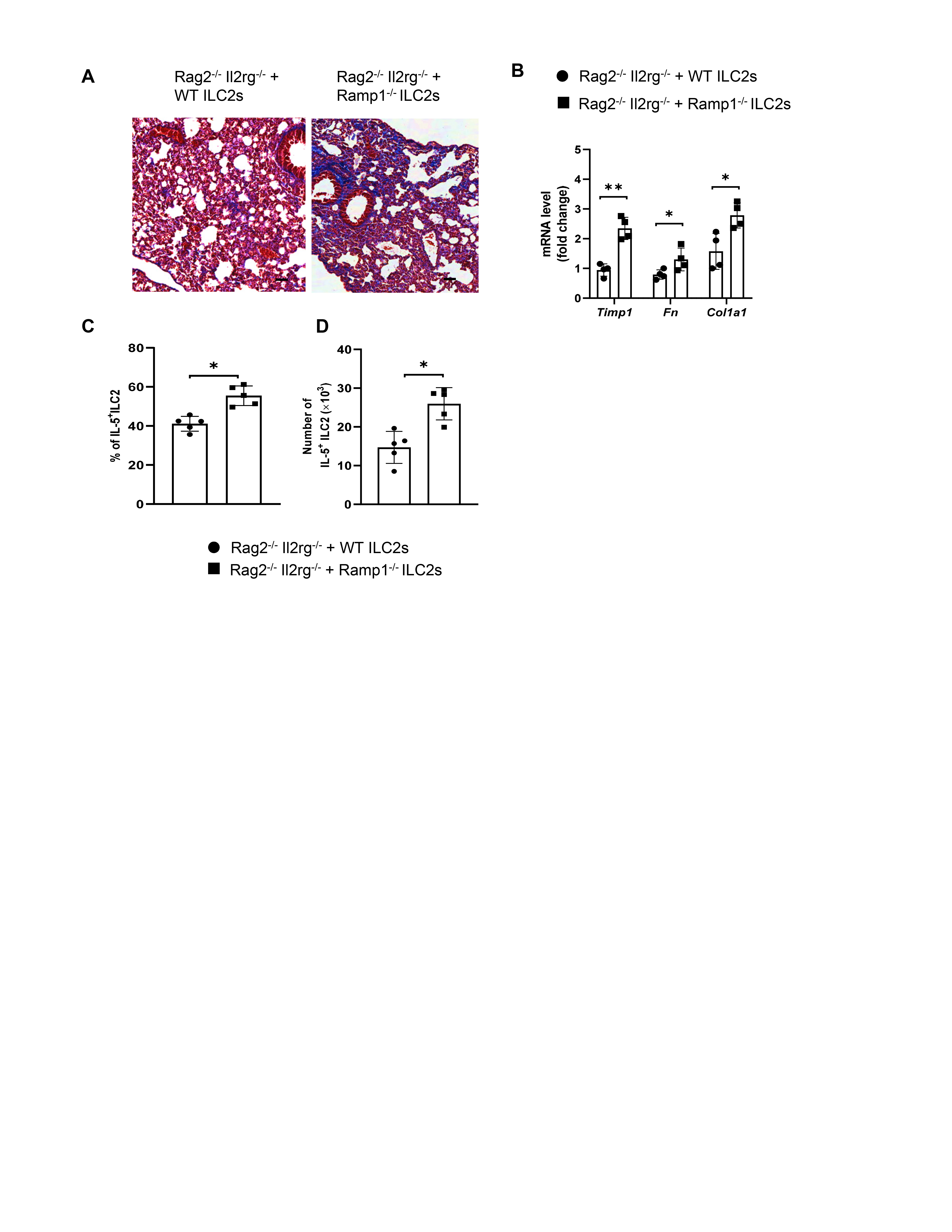

Supplement: Supplementary Figure 4 — CGRP- Ramp1 ILC2 axis regulates acute lung injury in a cell-intrinsic manner in alcohol-primed and bleomycin-treated mice. 2 × 10 (5) ILC2s were sorted from WT or Ramp1-/- mice and then transferred intravenously into Rag2-/- Il2rg-/- mice. After 7 days, alcohol diet was provided and bleomycin was administered intranasally to the recipient mice to induce pulmonary fibrosis. (A) Masson’s trichrome staining. (B) mRNA levels of the indicated genes related to fibrosis in lung. Fn: Fibronectin. (C, D) The frequency (C) and number (D) of IL-5+ ILC2s in whole lung tissues, determined by flow cytometry. Error bars Mean ± SEM; n =4, *p < 0.05, **p < 0.01. [file Image_4.jpeg]
